# Prenatal diagnosis of fetal unilateral lung agenesis complicated with cardiac malposition

**DOI:** 10.1186/1471-2393-13-79

**Published:** 2013-03-26

**Authors:** Ying Zhang, Miao Fan, Wei-dong Ren, Li-mei Xie, Chang-wei Ding, Wei Sun, Yu Wang, Ya-jun Guo, Ai-lu Cai

**Affiliations:** 1 Department of Sonography, Shengjing Hospital of China Medical University, No. 36 Sanhao Street, Heping District, Shenyang, 110004, China; 2 Department of Radiology, The First Affiliated Hospital of Sun Yat-sen University, Guangzhou, 510080, China; 3 Department of Radiology, Shengjing Hospital of China Medical University, No. 36 Sanhao Street, Heping District, Shenyang, 110004, China

**Keywords:** Fetus, Lung agenesis, Cardiac malposition, Fetal echocardiography

## Abstract

**Background:**

Fetal unilateral lung agenesis, complicated with cardiac shifting, is a rare anomaly, the diagnosis of which remains a challenge for many sonographers in routine screening programs. The present study describes a systematic approach for the diagnosis of fetal unilateral lung agenesis and cardiac malpositions in routine prenatal screening.

**Methods:**

A total of 18 cases of fetal unilateral lung agenesis complicated with cardiac malposition were reviewed. A systematic method was proposed to identify the fetal left side and right side according to the fetal head position and posture by acquiring a long axis and transverse view of the fetus. Fetal unilateral lung agenesis was diagnosed by evaluation of the ipsilateral pulmonary artery. The diagnosis was confirmed by postnatal echocardiography, digital radiology, and computed tomography after birth or by autopsy findings.

**Results:**

The left-sided fetal heart with the cardiac apex rotating to the left and posterior were confirmed in all 7 left lung agenesis cases, while the rightward shifting of the fetal heart together with the cardiac axis deviating to the right were confirmed in all 11 cases of right lung agenesis. The disappearance of the ipsilateral pulmonary artery was confirmed in all 18 cases of unilateral lung agenesis. Cardiac anomalies were present in a total of 7 of the18 cases of lung agenesis with 4 of 7 in cases of left lung agenesis and 3 of 11 in cases of right agenesis.

**Conclusions:**

The systematic approach introduced in the current report is helpful in the diagnosis of fetal unilateral lung agenesis complicated with cardiac malposition. The information provided by this study may be helpful to better understand unilateral lung agenesis anatomically and to facilitate its potential examination.

## Background

Prenatal diagnosis of cardiac malposition is one of the most challenging issues in obstetric sonography. This condition is often missed in routine screening programs due to the variable fetal positions in the uterus, particularly by inexperienced sonographers. Generally, fetal cardiac malposition can be classified into two main types [1,2]: One is caused by intrinsic congenital heart diseases, such as isolated dextrocardia, situs inversus, isolated levocardia, etc., while others are caused by extracardiac malformations such as unilateral lung agenesis, diaphragmatic hernia, etc. In this circumstance, the mediastinal shift leads to abnormal cardiac position in the thorax [3].

Lung agenesis is a rare developmental anomaly and prenatal diagnosis is difficult for many sonographers during routine screening. It is characterized by a complete absence of a lung and bronchi, and no blood supply to the affected side, either the left, the right, or bilateral side. It is often associated with a constellation of anomalies in other systems including the cardiovascular, gastrointestinal (tracheoesophageal fistula, imperforate anus), genitourinary, or skeletal (limb anomalies, vertebral segmentation anomalies) [4]. The estimated incidence is 1 per 10000–15000 autopsies [5].

The primary objective of the current study is to describe a systematic approach to diagnose fetal unilateral lung agenesis complicated with cardiac malposition.

## Methods

### Study population

A total of 5790 fetuses were examined via fetal echocardiography in our hospital in the time frame between May 2006 and March 2012. Routine fetal echocardiography was performed in all fetuses and the data saved as video clips. A total of 25 of the 5790 fetuses were lost to follow-up. Prenatal diagnosis in the others was confirmed by postnatal color Doppler echocardiography (CDE), digital radiography (DR), and computed tomography (CT) after birth or by autopsy. In total, 18 cases of fetal unilateral lung agenesis complicated with cardiac malposition were retrospectively reviewed in this study.

### Ethics approval

The study was approved by the Ethics Committee of Shengjing Hospital of China Medical University. Written informed consent was obtained from the parents for publication of clinical details, clinical images and videos.

### Assessment of fetal cardiac position

The patients were examined using ultrasound systems (Voluson 730 Expert and Voluson E8, GE Healthcare, Kretztechnik, Zipf, Austria), equipped with a 4–8 MHz transabdominal transducer.

**Figure 1:**
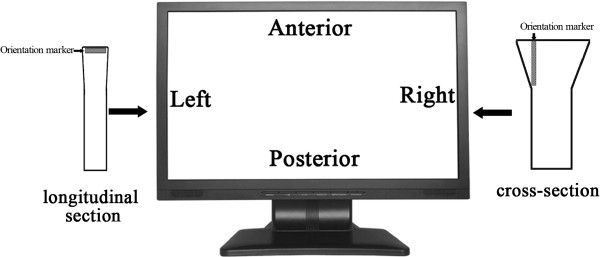
**The transducer orientation and display format of the image on the screen.** The orientation marker is depicted as the gray bar on the transducer. Transducer orientation marker indicates the direction that was displayed in the left side of the video screen. The direction of the beam on the screen is from top to bottom, which means the upper area of the screen displays the anterior part of the subject (from the perspective of the operator).

The technique used in this study to determine fetal cardiac position in the thorax relied on several parameters, including fetal head position relative to the maternal pelvis (cephalic, breech, or transverse lie) and posture (supine, prone, or side lie), transducer orientation, and display format of the image on the screen. The protocol followed in our study was as follows: the examiner was on the right side of the patient with the head of the patient on the same side as the ultrasound equipment. The transducer orientation marker indicates the direction that was displayed in the left side of the video screen and the direction of the beam on the screen is from top to bottom so that the upper area of the screen displayed the anterior part of the subject from the perspective of the operator, not the subject (Figure 1).

#### Step 1

**Figure 2:**
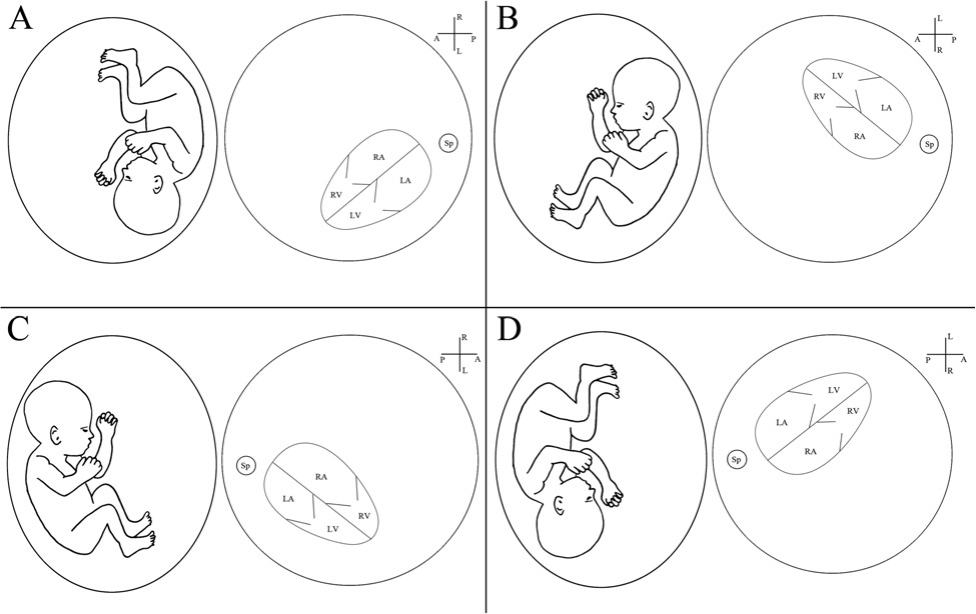
**Fetuses in various positions in uterus and the corresponding sonographic images from transverse plane at thorax level.** In panel **A** and **B**, the fetal spine is on the left side with the position of the fetus relative to the maternal pelvis is cephalic (panel **A**) or breech (panel **B**). In panel **C** and **D**, the fetal spine is on the right side with the position of the fetus relative to the maternal pelvis is breech (panel **C**) or cephalic (panel **D**). LA, left atrium; LV, left ventricle; RA, right atrium; RV, right ventricle; Sp, spine.

The first step was to determine fetal head position and posture. The transducer was oriented to in order to observe the long axis of the fetus. In circumstance showed in Figure 2A, the orientation marker of the transducer was directed toward the upper side so that the fetal head was displayed at the right side of the screen and the buttocks on the left side of the screen.

#### Step 2

The transducer was then rotated 90°counterclockwise from the initial position in order to see the transverse plane of the fetus. In circumstance showed in Figure 2A, the orientation marker of the transducer was directed toward the left side so that the spine was located on the right side of the screen, and the fetal abdominal or thorax area was on the left side of the screen. The upper area of the screen showed the right side of the fetus that was first examined by the beam; the lower area of the screen showed the left side of the fetus with the spine as the landmark. The position of the heart and gastric vacuole could then be established.

Figure 2B-D shows the fetal cardiac position in other common circumstances.

### Assessment of cardiac axis and bilateral lung

The fetal heart and the bilateral lung could be visualized in the transverse plane at the thorax level. Normally, the cardiac axis is about 45° on the left side. The ratio of the heart to the lung in the left and right is about 1:1:1 under normal circumstances.

### Assessment of structures of the heart and great vessels

In order to exclude fetal congenital heart disease after determination of the cardiac and visceral position, a four chamber view, the left and right outflow tract views, the three-vessel view, the aortic arch long axis view and the ductal arch view were acquired in all fetuses.

Under normal circumstances, the main pulmonary artery (MPA), the left pulmonary artery (LPA) and right pulmonary artery (RPA) could be visualized at the right outflow tract view. Pulmonary artery bifurcation is a characteristic anatomical structure that could be detected very easily.

## Results

**Figure 3:**
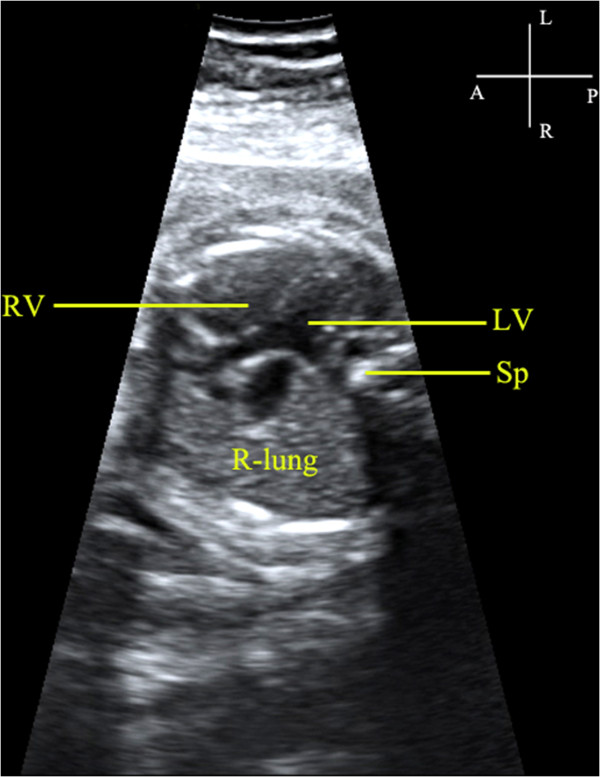
**Visualization of the position of the fetal heart**, **the right lung and the spine in the case of fetus at 27 gestational weeks diagnosed with left lung agenesis.** At the transverse plane of thorax level, the right lung was not detected and the fetal heart moved toward the left and posterior. The cardiac axis was about 120°toward left side. LV, left ventricle; RV, right ventricle; Sp, spine.

**Figure 4:**
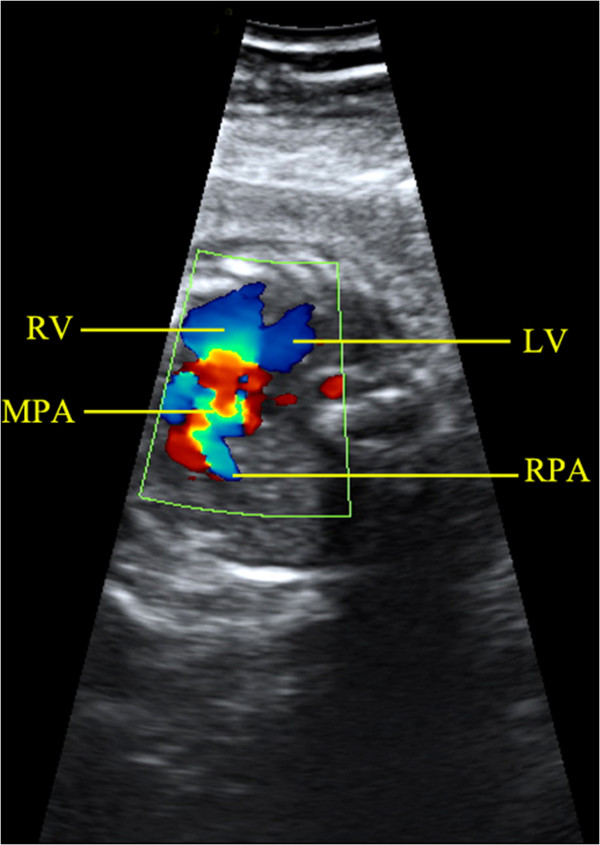
**Visualization of the pulmonary artery in the same fetus as Figure**3**with left lung agenesis.** The right pulmonary artery originates from the main pulmonary artery, while the left pulmonary artery was not detected. MPA, main pulmonary artery; LV, left ventricle; RPA, right pulmonary artery; RV, right ventricle.

**Figure 5:**
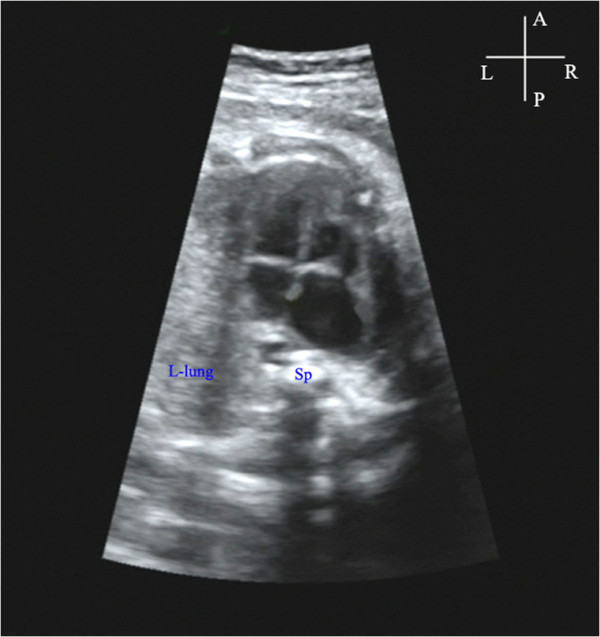
**Ultrasonographic image of a 35**-**gestational**-**week fetus diagnosed with right lung agenesis.** At the transverse plane of the thorax level, a right-sided heart beside the left lung was demonstrated and the cardiac axis was about 50° toward right side. Sp, spine.

The gestational age among the 18 cases of unilateral lung agenesis ranged from 18 to 35 (mean=28) weeks. The maternal age ranged from 25 to 37 (mean=32) years. Findings from the eighteen cases are summarized in Table 1. In cases of left lung agenesis, we confirmed that the fetal heart was on the left while the cardiac apex was rotated to the left and posterior at various angles (Figure 3). An additional movie file showed this in more detail [see Additional file 1: Video]. The MPA and RPA were detected however the LPA remained invisible in all the seven cases of left lung agenesis (Figure 4). We also provided an additional movie file to show more details [see Additional file 2: Video]. In the cases of the right lung agenesis, we confirmed that the fetal heart was shifted to the right with cardiac apex rotation to the right at various angles (Figure 5). The MPA and LPA were seen in ten cases and only the MPA could be detected in one case. The RPA remained invisible in all the eleven cases of right lung agenesis. Cardiac anomalies were present in a total of 7 of the18 cases of lung agenesis: 4 of 7 in cases of left lung agenesis and 3 of 11 in cases of right lung agenesis. All cases were confirmed by postnatal CDE, DR, and CT or by autopsy findings.

**Table 1:** Ultrasound appearance and outcomes of fetal unilateral lung agenesis with cardiac malposition in the current series

**Case**	**GA**	**Lung agenesis**	**Cardiac position**	**Cardiac axis**	**CHD**	**Visualization of the PA**	**Postnatal confirmation**
1	18	Left	Left	left axis 100°	None	MPA and RPA	Yes by CDE and DR
2	33	Left	Left	left axis 60°	None	MPA and RPA	Yes by CDE and CT
3	27	Left	Left	left axis 120°	None	MPA and RPA	Yes by autopsy
4	31	Left	Left	left axis 70°	Common ventricle and Primum ASD	MPA and RPA	Yes by autopsy
5	21	Left	Left	left axis 80°	DORV and VSD	MPA and RPA	Yes by autopsy
6	31	Left	Left	left axis 60°	VSD and TGA	MPA and RPA	Yes by autopsy
7	25	Left	Left	left axis 80°	VSD	MPA and RPA	Yes by CDE
8	26	Right	Right	right axis 50°	None	MPA and LPA	Yes by CDE, DR and CT
9	32	Right	Right	right axis 50°	None	MPA and LPA	Yes by CDE, DR, and CT
10	23	Right	Right	right axis 70°	None	MPA and LPA	Yes by CDE and DR
11	35	Right	Right	right axis 60°	None	MPA and LPA	Yes by CDE and CT
12	26	Right	Right	right axis 70°	None	MPA and LPA	Yes by CDE and CT
13	32	Right	Right	right axis 80°	None	MPA	Yes by CDE, DR and CT
14	35	Right	Right	right axis 50°	None	MPA and LPA	Yes by CDE, DR and CT
15	22	Right	Right	right axis 80°	None	MPA and LPA	Yes by CDE and CT
16	35	Right	Right	right axis 60°	AVSD	MPA and LPA	Yes by autopsy
17	27	Right	Right	right axis 60°	ccTGA and VSD	MPA and LPA	Yes by autopsy
18	28	Right	Right	right axis 60°	TGA and VSD	MPA and LPA	Yes by autopsy

*ASD*, atrial septal defect; *AVSD*, atrioventricular septal defect; *ccTGA*, congenital corrected transposition of the great arteries; *CDE*, color Doppler echocardiography; *CHD*, congenital heart disease; *CT*, computed tomography; *DORV*, double outlet right ventricle; *DR*, digital radiography; *GA*, gestational age at examination (in weeks); *LPA*, left pulmonary artery; *MPA*, main pulmonary artery; *PA*, pulmonary artery; *RPA*, right pulmonary artery; *TGA*, transposition of the great arteries; *VSD*, ventricular septal defect.

**Figure 6:**
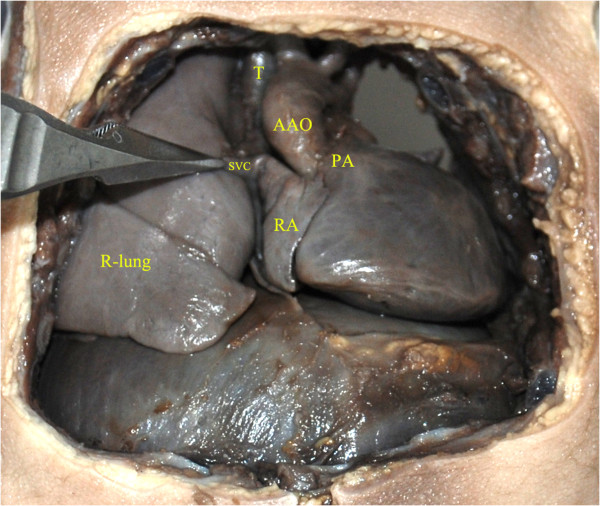
**Autopsy findings of fetal**, **left lung agenesis complicated with cardiac malposition.** The anterior chest wall, the thymus, and the pericardium were removed to demonstrate the shape and location of the heart. The posterior chest wall in the left side was also removed to confirm any anomalies behind the heart. The fetal heart occupied almost the whole hemithorax at the left side with the cardiac apex rotated toward the left and posterior. The right lung was normal and the left lung not seen. AAO, ascending aorta; PA, pulmonary artery; RA, right atrium; SVC, superior vena cava; T, trachea.

**Figure 7:**
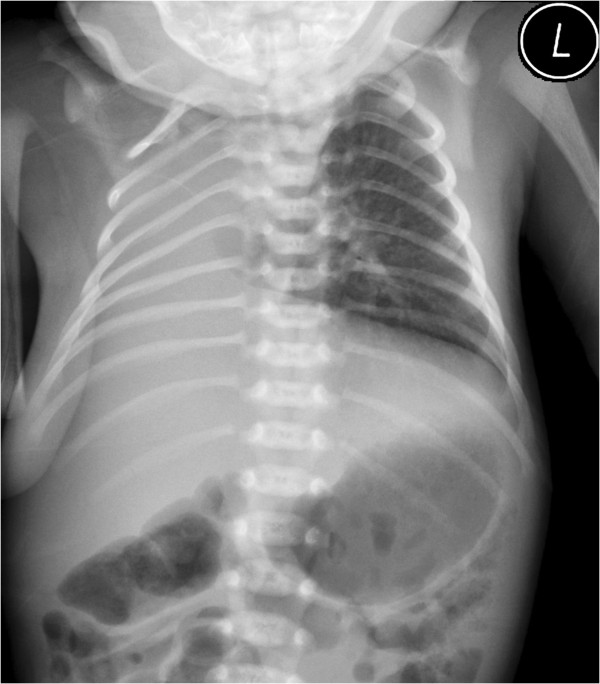
**Digital radiographs of the chest showing an opaque**, **right hemithorax with mediastional shift to the left in a neonate that was diagnosed with right lung agenesis at 28 gestational weeks.**

**Figure 8:**
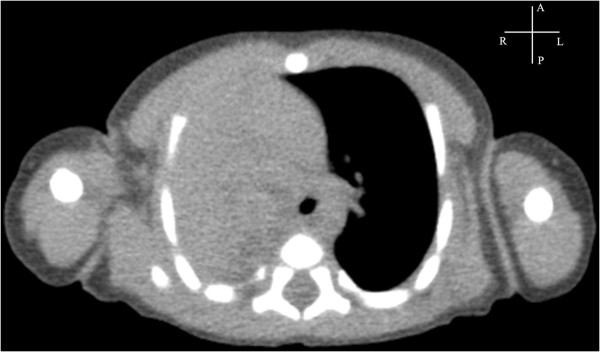
**Computed tomography of the same neonate in Figure**7**showing right lung agenesis.**

Figure 6 shows the autopsy findings in a case of left lung agenesis with cardiac malposition (the fetus died of intrauterine infection at 32 gestational weeks). Figures 7 and 8 shows the postnatal DR and CT images in a case of right lung agenesis. The cardiac position and spatial relationships of the heart and lung are shown clearly.

## Discussion

The specificity and sensitivity of diagnosis by fetal echocardiography has improved greatly in the last twenty years due to the advances in technology and increased experience of the prenatal sonographers [6,7] and most fetal congenital heart diseases could be diagnosed correctly by experienced examiners. However, fetal cardiac malposition remains a great challenge for most sonographers performing routine prenatal ultrasound due to constant movement of the fetuses. Many screening sonographers always establish the left/right orientation of the fetus on the basis of the position of internal organs, such as stomach or heart. It is certainly incorrect as the position of internal organs may vary from the normal. More over, anomalies of laterality often affect both stomach and the heart (e.g., situs inversus). Bronshtein et al. (2002) proposed an easy right-hand role to define fetal situs [8]. The Cordes technique has also been reported to determine fetal right/left axis using easily identified anatomic landmarks [9,10]. These techniques are certainly of great value in evaluating fetal situs and only require following some simple rules, though the examiner may be unclear as to why and how the rules work. In our report, we did not propose a novel technique but we did present a systematic way to identify the fetal left and right sides, according to the fetal head position and posture, by merely acquiring long axis and transverse views of the fetus. In the present study, the time requirement for determination of fetal heart position was about 3–5 s for an experienced sonographer. The minimal time required to obtain results is another benefit of our method.

Unilateral lung agenesis, an extremely rare anomaly in which one of the lungs and its associated bronchus and vessels are absent, results due to the failure of origin of lung bud from tracheo-bronchial tree. The lack of fullness of the chest, the crowding of ribs, and the displacement of the mediastinum, usually toward the affected side, is often found in these patients [11]. This can be easily diagnosed after birth by X-ray and CT though prenatal diagnosis remains difficult [12].

Fetal cardiomediastinal shift is generally caused by extra-cardiac situations such as congenital diaphragmatic hernia, cystic lung malformations, and unilateral lung agenesis, etc. [13,14]. The fetal heart can potentially move to any position in thorax under these circumstances. Cardiomediastinal shift may often be the first clue suggesting the possibility of unilateral lung agenesis. Since the fetal heart, the left lung and the right lung can be detected in the transverse plane at the thorax level, the possibility of unilateral lung agenesis should be suspected in the case of invisible lung tissue in left or right hemithorax combined with cardiac shifting to the ipsilateral thorax [15,16]. However, this condition is difficult to diagnose for most screening sonographers as the large sized thymus in the fetus may be mistaken for lung tissue.

The MPA, as well as the LPA and RPA can routinely be detected clearly by CDE in second trimester screening examinations. A fundamental aspect of the diagnosis is the inability to detect the pulmonary artery bifurcation and the LPA or RPA. In our reports, the LPA was not visible in all cases of left lung agenesis while the RPA remained invisible in all cases of right lung agenesis. Previous studies have reported that the ipsilateral pulmonary veins were also invisible in the case of unilateral lung agenesis [14]. Nonetheless, in the current study it appears that the invisibility of the ipsilateral pulmonary artery played a more important role in the diagnosis of unilateral lung agenesis since the larger, higher-velocity pulmonary artery can be detected more easily than the smaller, lower-velocity pulmonary veins.

Lung agenesis is an extremely rare malformation and has mostly been reported in case reports or literature reviews. We reported 18 cases in our report, both the left and right lung agenesis, all with prenatal and postnatal clinical data. The relatively large number of this rare case, together with the prenatal and postnatal data, is a benefit of our research. Differed from previous reports, we also evaluated cardiac axis deviation in each of the 18 cases, which is another highlight of our study. The cardiac axis was rotated in all cases in our report with the cardiac apex rotated rightward or leftward at various angles depending on whether the case was right or left lung agenesis. This could be considered a useful indirect sign of the anomaly since the cardiac axis deviation can be visualized easily.

Previous studies have demonstrated that lung agenesis is frequently associated with a constellation of other anomalies, particularly cardiovascular malformations. According to the literature, associated cardiac anomalies with unilateral lung agenesis varied greatly. In the review by Maltz and Nadas (1968), cardiac and vascular defects were noted in 23% of the 156 reported patients [17]. A more recent review [18] by Meller et al. (2012) suggested that left lung agenesis was more frequently isolated than right lung agenesis. They found that only 1 in 6 cases of left lung agenesis had a cardiovascular anomaly, whereas 19 of 25 cases with right-sided presented with a cardiovascular anomaly, the most common of which was scimitar syndrome (15 cases). They also suggested a better outcome for those with left lung compared to those with right lung agenesis. A postnatal study [19] by Chou et al. (2007) suggested that cardiovascular disorders were quite common (11 in 14 cases) especially in cases of right lung agenesis (9 in 10 cases), though some complex cardiac malformations were associated with left lung agenesis. The associated intracardiac defects involved ventricular septal defect, atrial septal defect, persistent left superior vena cava, and some complex cyanotic heart diseases (double outlet right ventricle, atrioventricular septal defect, and pulmonary stenosis). In our report, no scimitar syndrome was found in the associated malformations and the proportion and nature of associated cardiac malformations of unilateral lung agenesis is not fully consistent with previous reports. This may be due to the very small number of cases involving this rare malformation.

The prognosis of the patients with unilateral lung agenesis has improved dramatically in the past decade due to the advances in prenatal diagnosis and neonatal care [20]–[22]. Correct prenatal diagnosis will not only offer parents a better understanding of the congenital abnormality through enhanced prenatal counseling, but will also provide a large amount of information for neonatal physicians that could reduce the mortality and potentially improve the prognosis.

## Conclusion

The current study introduces a systematic approach for the diagnosis and characterization of fetal unilateral lung agenesis complicated with cardiac malposition. This approach may be helpful to better understanding the anomaly in anatomy and facilitate the examination for and diagnosis of this rare malformation.

### Consent

Written informed consent for publication of clinical details, clinical images and videos was obtained from the parents.

## Competing interests

The authors declare that they have no competing interests.

## Authors’ contributions

YZ, MF and W-dR designed the study, performed some of the fetal echocardiography and drafted the manuscript. L-mX, WS, YW, Y-jG and A-lC collected the clinical data and performed some of the fetal echocardiography. C-wD performed radiographic examinations. All authors read and approved the final manuscript.

## Pre-publication history

The pre-publication history for this paper can be accessed here:

http://www.biomedcentral.com/1471-2393/13/79/prepub

## Supplementary Material

Additional file 1: Video. Visualization of the position of the fetal heart, the right lung and the spine in the case of fetus at 27 gestational weeks diagnosed with left lung agenesis. At the transverse plane of thorax level, the right lung was not detected and the fetal heart moved toward the left and posterior. The cardiac axis was about 120°toward left side. Click here for file

Additional file 2: Video. Visualization of the pulmonary artery in the case of fetus at 27 gestational weeks diagnosed with left lung agenesis. The right pulmonary artery originates from the main pulmonary artery, while the left pulmonary artery was not detected. Click here for file

## References

[B1] Comstock CH Smith R Lee W Kirk JS Right fetal cardiac axis: clinical significance and associated findings Obstet Gynecol 1998 91 495 499 10.1016/S0029-7844(98)00018-0 9540929

[B2] Smith RS Comstock CH Kirk JS Lee W Ultrasonographic left cardiac axis deviation: a marker for fetal anomalies Obstet Gynecol 1995 85 187 191 10.1016/0029-7844(94)00350-M 7824228

[B3] Allan LD Lockhart S Intrathoracic cardiac position in the fetus Ultrasound Obstet Gynecol 1993 3 93 96 10.1046/j.1469-0705.1993.03020093.x 12797299

[B4] Biyyam DR Chapman T Ferguson MR Deutsch G Dighe MK Congenital lung abnormalities: embryologic features, prenatal diagnosis, and postnatal radiologic-pathologic correlation Radiographics 2010 30 1721 1738 10.1148/rg.306105508 21071385

[B5] Malcon MC Malcon CM Cavada MN Caruso PE Real LF Unilateral pulmonary agenesis J Bras Pneumol 2012 38 526 529 10.1590/S1806-37132012000400016 22964938

[B6] Mohammed NB Chinnaiya A Evolution of foetal echocardiography as a screening tool for prenatal diagnosis of congenital heart disease J Pak Med Assoc 2011 61 904 909 22360034

[B7] Yagel S Cohen SM Messing B Valsky DV Three-dimensional and four-dimensional ultrasound applications in fetal medicine Curr Opin Obstet Gynecol 2009 21 167 174 10.1097/GCO.0b013e328329243c 19996869

[B8] Bronshtein M Gover A Zimmer EZ Sonographic definition of the fetal situs Obstet Gynecol 2002 99 1129 1130 10.1016/S0029-7844(02)02017-3 12052611

[B9] Cordes TM O'Leary PW Seward JB Hagler DJ Distinguishing right from left: a standardized technique for fetal echocardiography J Am Soc Echocardiogr 1994 7 47 53 8155333 10.1016/s0894-7317(14)80417-3

[B10] Ozkutlu S Bostan OM Deren O Onderoğlu L Kale G Güçer S Orhan D Prenatal echocardiographic diagnosis of cardiac right/left axis and malpositions according to standardized Cordes technique Anadolu Kardiyol Derg 2011 11 131 136 21303758 10.5152/akd.2011.033

[B11] Ratan SK Grover SB Lung agenesis in a neonate presenting with contralateral mediastinal shift Am J Perinatol 2001 18 441 446 10.1055/s-2001-18788 11733859

[B12] Epelman M Kreiger PA Servaes S Victoria T Hellinger JC Current imaging of prenatally diagnosed congenital lung lesions Semin Ultrasound CT MR 2010 31 141 157 10.1053/j.sult.2010.01.002 20304322

[B13] Yancey MK Richards DS Antenatal sonographic findings associated with unilateral pulmonary agenesis Obstet Gynecol 1993 81 847 849 8469495

[B14] Kalache KD Chaoui R Paris S Bollmann R Prenatal diagnosis of right lung agenesis using color Doppler and magnetic resonance imaging Fetal Diagn Ther 1997 12 360 362 10.1159/000264506 9475368

[B15] Roberts A Prenatal diagnosis of pulmonary hypoplasia Prenat Diagn 2001 21 304 307 10.1002/pd.45 11288122

[B16] Levine D Barnewolt CE Mehta TS Trop I Estroff J Wong G Fetal thoracic abnormalities: MR imaging Radiology 2003 228 379 388 10.1148/radiol.2282020604 12821772

[B17] Maltz DL Nadas AS Agenesis of the lung. Presentation of eight new cases and review of the literature Pediatrics 1968 42 175 188 5657673

[B18] Meller CH Morris RK Desai T Kilby MD Prenatal diagnosis of isolated right pulmonary agenesis using sonography alone: case study and systematic literature review J Ultrasound Med 2012 31 2017 2023 23197556 10.7863/jum.2012.31.12.2017

[B19] Chou AK Huang SC Chen SJ Huang PM Wang JK Wu MH Chen YS Chang CI Chiu IS Wu ET Unilateral lung agenesis–detrimental roles of surrounding vessels Pediatr Pulmonol 2007 42 242 248 10.1002/ppul.20561 17238192

[B20] Hourrier S Salomon LJ Bault JP Dumez Y Ville Y Prenatal diagnosis and management of foetal lung lesions Rev Mal Respir 2011 28 1017 1024 10.1016/j.rmr.2011.09.002 22099407

[B21] Backer CL Kelle AM Mavroudis C Rigsby CK Kaushal S Holinger LD Tracheal reconstruction in children with unilateral lung agenesis or severe hypoplasia Ann Thorac Surg 2009 88 624 630 10.1016/j.athoracsur.2009.04.111 19632424

[B22] Achiron R Zalel Y Lipitz S Hegesh J Mazkereth R Kuint J Jacobson J Yagel S Fetal lung dysplasia: clinical outcome based on a new classification system Ultrasound Obstet Gynecol 2004 24 127 133 10.1002/uog.1112 15287048

